# *n*-Butylidenephthalide Protects against Dopaminergic Neuron Degeneration and α-Synuclein Accumulation in *Caenorhabditis elegans* Models of Parkinson's Disease

**DOI:** 10.1371/journal.pone.0085305

**Published:** 2014-01-08

**Authors:** Ru-Huei Fu, Horng-Jyh Harn, Shih-Ping Liu, Chang-Shi Chen, Wen-Lin Chang, Yue-Mi Chen, Jing-En Huang, Rong-Jhu Li, Sung-Yu Tsai, Huey-Shan Hung, Woei-Cherng Shyu, Shinn-Zong Lin, Yu-Chi Wang

**Affiliations:** 1 Graduate Institute of Immunology, China Medical University, Taichung, Taiwan; 2 Center for Neuropsychiatry, China Medical University Hospital, Taichung, Taiwan; 3 Department of Pathology, China Medical University Hospital, Taichung, Taiwan; 4 Graduate Institute of Basic Medical Science, China Medical University, Taichung, Taiwan; 5 Department of Biochemistry and Molecular Biology, National Cheng Kung University, Tainan, Taiwan; 6 Department of Neurosurgery, China Medical University Beigang Hospital, Yunlin, Taiwan; 7 Department of Neurosurgery, Tainan Municipal An-Nan Hospital-China Medical University, Tainan, Taiwan; 8 Biomedical Technology and Device Research Laboratories, Industrial Technology Research Institute, Hsinchu, Taiwan; Brown University/Harvard, United States of America

## Abstract

**Background:**

Parkinson's disease (PD) is the second most common degenerative disorder of the central nervous system that impairs motor skills and cognitive function. To date, the disease has no effective therapies. The identification of new drugs that provide benefit in arresting the decline seen in PD patients is the focus of much recent study. However, the lengthy time frame for the progression of neurodegeneration in PD increases both the time and cost of examining potential therapeutic compounds in mammalian models. An alternative is to first evaluate the efficacy of compounds in *Caenorhabditis elegans* models, which reduces examination time from months to days. *n*-Butylidenephthalide is the naturally-occurring component derived from the chloroform extract of *Angelica sinensis*. It has been shown to have anti-tumor and anti-inflammatory properties, but no reports have yet described the effects of *n*-butylidenephthalide on PD. The aim of this study was to assess the potential for *n*-butylidenephthalide to improve PD in *C. elegans* models.

**Methodology/Principal Findings:**

In the current study, we employed a pharmacological strain that expresses green fluorescent protein specifically in dopaminergic neurons (BZ555) and a transgenic strain that expresses human α-synuclein in muscle cells (OW13) to investigate the antiparkinsonian activities of *n*-butylidenephthalide. Our results demonstrate that in PD animal models, *n*-butylidenephthalide significantly attenuates dopaminergic neuron degeneration induced by 6-hydroxydopamine; reduces α-synuclein accumulation; recovers lipid content, food-sensing behavior, and dopamine levels; and prolongs life-span of 6-hydroxydopamine treatment, thus revealing its potential as a possible antiparkinsonian drug. *n*-Butylidenephthalide may exert its effects by blocking *egl-1* expression to inhibit apoptosis pathways and by raising *rpn-6* expression to enhance the activity of proteasomes.

**Conclusions/Significance:**

*n*-Butylidenephthalide may be one of the effective neuroprotective agents for PD.

## Introduction

Parkinson's disease (PD) is the second most common disorder of the central nervous system, the incidence of which quickly augments in the people over the age of 60 years. The apparent symptoms of PD are movement disorders, including muscle rigidity, bradykinesia, and tremors. Nevertheless, behavioral and cognitive problems, including dementia, depression, anxiety, and sleep disturbances, are also involved in the later stages of the disease [1]. Two clinical-pathological findings describe both familial and sporadic PD: the development of Lewy bodies (aggregation of α-synuclein) in the brain tissue and the selective loss of midbrain dopaminergic (DA) neurons in the substantia nigra [2]. The reason of PD remains unclear, but it most likely results from complex interactions between a number of genetic and environmental factors [3].

PD is associated with ageing and its progression, and a raise in free radicals has been suggested as the fundamental factor. 6-hydroxydopamine (6-OHDA) is a neurotoxin that is thought to enter neurons via dopamine reuptake transporters and is used to selectively destroy DA neurons and then reduce dopamine levels *in vivo* treatment. The main use for 6-OHDA in medical research is to induce experimental parkinsonism in laboratory animals to establish and test new medicines for treating PD in humans [4]. The α-synuclein protein is encoded by the *SNCA* gene (*PARK1*) and is abundant in the human brain. In PD, the accumulation of α-synuclein is a critical challenge to DA neurons, especially as they aggregate in inclusions and destroy the cellular machinery required for their degradation. These influences may be compounded by PD-related dysregulation of chaperones [5], a continually accelerating loss in general cellular homeostasis [6], [7]. Some reports also have implied that α-synuclein associates with the negatively charged surface of phospholipids and participates membrane composition and turnover [8], [9]. The interacting of α-synuclein with lipid membranes changes the phospholipid bilayer structure, and leads to the formation of small vesicles. Moreover, the toxic property of these protein aggregates tends to promote lipid peroxidation in the tissues via a raise in the reactive oxygen species load [10].

**Figure 1:**
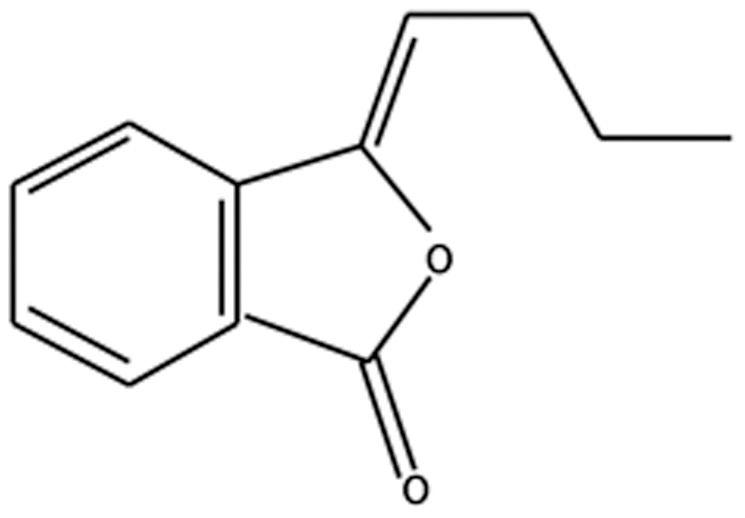
Chemical structure of *n*-butylidenephthalide.

At present there is no effective cure for PD. Obtainable drugs, including levodopa, coenzyme Q10, creatine, dopamine agonists (ropinirole and pramipexole), and monoamine oxidase B inhibitors (rasagiline and selegiline), only delay and relieve the symptoms [11]. The most hopeful therapeutic route for PD involves the finding of small molecules that can improve the disease as well as prevent its symptoms. Many herbs in China have been found to be valuable neuroprotective agents. *Angelica sinensis*, commonly called "dong quai", is a biennial and perennial herb that is distributed in scattered locations across China and Japan. The radix of *Angelica sinensis* is officially recorded in the *Chinese Pharmacopoeia* [12]. It has been available for treatments such as anemia, female irregular menstruation, cardiovascular disease, amenorrhoea, hypertension, chronic bronchitis, asthma, and rheumatism [12]. *n*-Butylidenephthalide (Figure 1) is the key bioactive component derived from the chloroform extract of *Angelica sinensis* herbs, has been confirmed to have a variety of potential pharmacological activities, such as anti-cancer [13]–[15], anti-angiogenesis [16], anti-inflammatory [17], anti-platelet [18], vasorelaxant [19]–[21], anti-anginal [22], [23], and anti-atherosclerotic [24] effects. Here, we study that *n*-butylidenephthalide could be examined as a prophylactic as well as an adjuvant agent for its advantageous effects on PD.

Drug development for PD is a main challenge. Testing the therapeutic value of candidate compounds in vertebrate disease models requires time-consuming and costly experimental studies. The establishment of rapid, simple and inexpensive *in vivo* assays to assess the small molecule is therefore of foremost importance. Utilizing the simple well-studied nematode *Caenorhabditis elegans* as an animal model system affords several advantages in the study of PD. (1) This animal is reasonably small, has a short life cycle, and is inexpensive to grow in liquid culture. Large-scale analysis is possible. (2) It has 8 DA neurons, with completely mapped neuronal networks [25]. (3) It also has PD-related homologous gene, and the pathways involved in the function and metabolism of the DA neurons have been well conserved through evolution [26]–[41]. (4) Specific behavioral responses in this animal are well-known to be connected to DA signaling [42], [43]. (5) The large number of mutant strains are available and the transgenic/knockdown methods can be easily operated [44]. (6) This animal is wholly transparent; DA neurons can be directly observed through the expression of a fluorescent protein [45], [46], and a transgenic strain that expresses human α-synuclein-fluorescent protein fusion proteins can be used to estimate the amount of α-synuclein accumulation [47]–[50]. (7) It is convenient to use neurotoxins, including 6-OHDA and 1-methyl-4-phenyl pyridinium, to induce DA neuron degeneration in this animal, thus producing a useful pharmacological model of PD [51]–[53]. Here, we used the *C. elegans* animal model system to evaluate the effects of *n*-butylidenephthalide on PD and to study the potential mechanism of drug action.

## Materials and Methods

### Strains, culture and synchronization

*C. elegans* of wild-type Bristol N2, transgenic BZ555 (Pdat-1:GFP; GFP expressed specifically in dopaminergic neurons) and transgenic OW13 (Punc-54:α-synuclein:YFP+unc-119; human α-synuclein protein fused to YFP expressed specifically in body wall muscles) were provided by the Caenorhabditis Genetics Center (University of Minnesota). On the basis of previous standard protocols [54], we cultured the animals on nematode growth medium (NGM) plates seeded with the *Escherichia coli* strain OP50 or HB101 as food sources (OP50 for compound efficacy analyses and HB101 for food clearance tests) at 22°C. Fertilized eggs (embryos) were isolated from gravid adults by hypochlorite treatment (2% sodium hypochlorite and 0.5 M NaOH). After 20 h incubation at 22°C in M9 buffer to collect synchronized L1 larvae, the animals were transferred to OP50/NGM plates and then incubated for 24 h at 22°C to obtain L3 larvae.

### Food clearance test

Synthesized *n*-butylidenephthalide (mol. wt. 188.23, 95% purity) was purchased from Lancaster Synthesis Ltd. (Newgate Morecambe, UK), dissolved in dimethyl sulfoxide (DMSO) to 1 M, and stored at −20°C as a master stock solution. A food clearance test was used to determine the impact of *n*-butylidenephthalide on *C. elegans* physiology [55], [56]. A culture of *E. coli* was grown overnight and then resuspended at a final optical density (OD) of 6.6 in nematode S-medium [56]. *n*-Butylidenephthalide was diluted into the *E. coli* suspension to the desired concentrations. The final concentration of DMSO in all *n*-butylidenephthalide-treated cultures was 2% (*v/v*). Fifty microliters of the final mixture was loaded per well in a 96-well plate. Approximately 20–30 synchronized L1 animals in 10 µl of S-medium were added to an *E. coli* suspension containing a series of concentrations of *n*-butylidenephthalide and incubated in a 96-well microtiter plate at 25°C. The absorbance (OD 595 nm) of the culture was determined every day for 6 days using a SpectraMax M2 Microplate Reader (Molecular Devices, Silicon Valley, CA).

### 6-OHDA and *n*-butylidenephthalide treatment

Animals were treated with 6-OHDA (Sigma, St. Louis, MI) in order to induce selective degeneration of DA neurons [57]. In brief, 50 mM 6-OHDA and 10 mM ascorbic acid were added to OP50/S-medium mix with *n*-butylidenephthalide. The final concentration of DMSO in all treated cultures was 1% (*v/v*). Synchronized L3 larvae were then transferred onto the treated cultures, incubated for 1 h at 22°C and mixed gently every 10 min. After 1 h of treatment, animals were washed three times with M9 buffer and then incubated in OP50/NGM plates with *n*-butylidenephthalide. After 24 h, animals were transferred to OP50/NGM plates containing *n*-butylidenephthalide and 0.04 mg/mL 5-fluoro-2′-deoxyuridine (FUDR, Sigma, St. Louis, MI) to reduce the production of progeny. Animals were scored with various assays 72 h after treatment.

### Quantitative assay of dopaminergic neurodegeneration

Assay of dopaminergic neurodegeneration was performed in animals treated with 6-OHDA or *n*-butylidenephthalide/6-OHDA, as described previously. After 72 h of treatment at 22°C, BZ555 animals were washed three times with M9 buffer, and then mounted onto a 2% agar pad on a glass slide using 100 mM sodium azide (Sigma, St. Louis, MI) and enclosed with a coverslip. Imaging of immobilized animals was carried out with an Axio Observer inverted fluorescence microscope (Carl Zeiss MicroImaging GmbH, Göttingen Germany). Fluorescence intensity was estimated using AxioVision software (Carl Zeiss, Göttingen, Germany).

### Quantitative assay of α-synuclein accumulation

Accumulation of α-synuclein protein was assayed in control and *n*-butylidenephthalide-treated OW13 animals. Synchronized OW13 L3 larvae were cultured on OP50/NGM plates containing 0.04 mg/mL FUDR (Sigma, St. Louis, MI) and *n*-butylidenephthalide for 72 h at 22°C, then washed three times using M9 buffer and transferred to 2% agarose pads on glass slides, mounted with 100 mM sodium azide and enclosed with a coverslip. Immobilized animals were imaged on an Axio Observer inverted fluorescence microscope (Carl Zeiss, Germany) to monitor the accumulation of α-synuclein protein, and accumulation was estimated using AxioVision software by measuring fluorescence intensity.

### Quantitative assay of lipid deposits

Nile red (Invitrogen, Carlsbad, CA) is a fluorescent stain specific for to detect intracellular lipid droplets. The stock solution was prepared by dissolving 0.5 mg Nile red dye in 1 mL of acetone, which was then mixed with *E. coli* OP50 suspension in the ratio 1∶250 as described previously [51]. Synchronized OW13 L3 larvae were cultured on Nile red/OP50/NGM plates containing 0.04 mg/mL FUDR and *n*-butylidenephthalide for 72 h at 22°C. Animals were washed and mounted onto 2% agarose pads using 100 mM sodium azide and enclosed with a coverslip. Immobilized animals were imaged on an Axio Observer inverted fluorescence microscope (Carl Zeiss, Germany) to monitor the lipid deposits. Fat staining was estimated using AxioVision software by measuring fluorescence intensity.

### Food-sensing behavior test

The food-sensing behavior test was carried out by evaluating the function of *C. elegans* DA neurons [58], [59]. Briefly, test plates were prepared by spreading *E. coli* overnight at 37°C in a ring with an inner diameter of 1 cm and an outer diameter of 8 cm on 9-cm diameter NGM agar plates to avoid the animals reaching the edge of the plate during the test. Well fed 6-OHDA-treated or *n*-butylidenephthalide/6-OHDA-treated adult animals were washed with M9 buffer and then transferred to the center of an assay plate with or without bacterial lawn in a drop of M9 buffer. Five minutes after transfer, the locomotory rate of each animal was measured in 20-s intervals. The slowing rate was estimated as the percentage of the locomotory rate in the bacteria lawn compared with that in the no bacteria lawn. The average slowing rate among 10 animals was defined as the result of each analysis. In all analyses, plates were numbered so that the experimenter was blind to the treatment of the animal.

### Dopamine content analysis

The dopamine content in animals treated with 6-OHDA or *n*-butylidenephthalide/6-OHDA was evaluated by high-performance liquid chromatography (HPLC) analysis combined with a chemiluminescence reaction assay as described previously [59]. Animals were washed with M9 buffer, and were then temporarily frozen in liquid nitrogen. For the preparation of the extract, frozen animal pellets were resuspended in buffer A (2 mM EDTA, 200 mM perchloric acid, 2 mM sodium metabisulfite), sonicated and centrifuged at 10,000 *g* for 5 min. The supernatants were analyzed by HPLC.

### Life-span measurement

A Life-span examine was carried out by transferring control, 6-OHDA-treated and *n*-butylidenephthalide/6-OHDA-treated adult animals every 3 days to a fresh control or treated plate. A total of 0.04 mg/mL of FUDR was added to each plate to diminish the production of progeny. The number of live, dead and missing animals was counted each day until the last animal was dead. Experiments were performed by three different experimenters. Survival curves were plotted by the product-limit method of Kaplan and Meier; statistical analyses were carried out by SPSS software.

### Quantitative real-time PCR

Total RNA was isolated from synchronized control or experimental adult animals using TRIzol reagent (Invitrogen, Carlsbad, CA) according to the manufacturer's instructions. cDNA was synthesized using the SuperScript One-Step RT-PCR system (Invitrogen, Carlsbad, CA). SYBR Green real-time qPCR assays were carried out with a 1∶20 dilution of cDNA using an ABI StepOnePlus system (Applied Biosystems). Data were calculated with the comparative 2ΔΔ*C*_t_ method using the geometric mean of *cdc-42*, *pmp-3* and *Y45F10D.4* as the endogenous control [60]. Table S1 shows details of the primers used in the current study [61].

### 26S proteasome activity analysis

*In vitro* 26S proteasome activity analyses were carried out as previously described [61]. Briefly, using a Precellys 24 homogenizer (Bertin Technologies, Montigny-le-Bretonneux, France), animals were lysed using proteasome activity assay buffer containing 50 mM Tris-HCl (Ph 7.5), 250 mM sucrose, 5 mM MgCl_2_, 2 mM ATP, 1 mM dithiothreitol and 0.5 mM EDTA. The lysate was centrifuged at 10,000 *g* for 15 min at 4°C. For each assay, 25 µg of total lysate was loaded into each well of a 96-well microtiter plate, after which fluorogenic substrate was added. For determining the chymotrypsin-like activity of the proteasome, Z-Gly-Gly-Leu-AMC (Enzo Life Sciences, Farmingdale, NY) was used as a substrate. After incubation for 1 h at 25°C, fluorescence (an excitation wavelength 380 nm and an emission wavelength 460 nm) was measured with a SpectraMax M2 Microplate Reader (Molecular Devices, Silicon Valley, CA).

### Statistics

Statistical analyses are shown as mean ± standard deviation (SD) from independent experiments. Three replicates were done of each experiment. The differences among groups were determined by One-way ANOVA analysis followed by a Newman-Keuls *post hoc* test. Values of *p*<0.05 were determined to be statistically significant.

## Results

### Determining the *n*-butylidenephthalide concentration range for treatment of *C. elegans* by food clearance test

**Figure 2:**
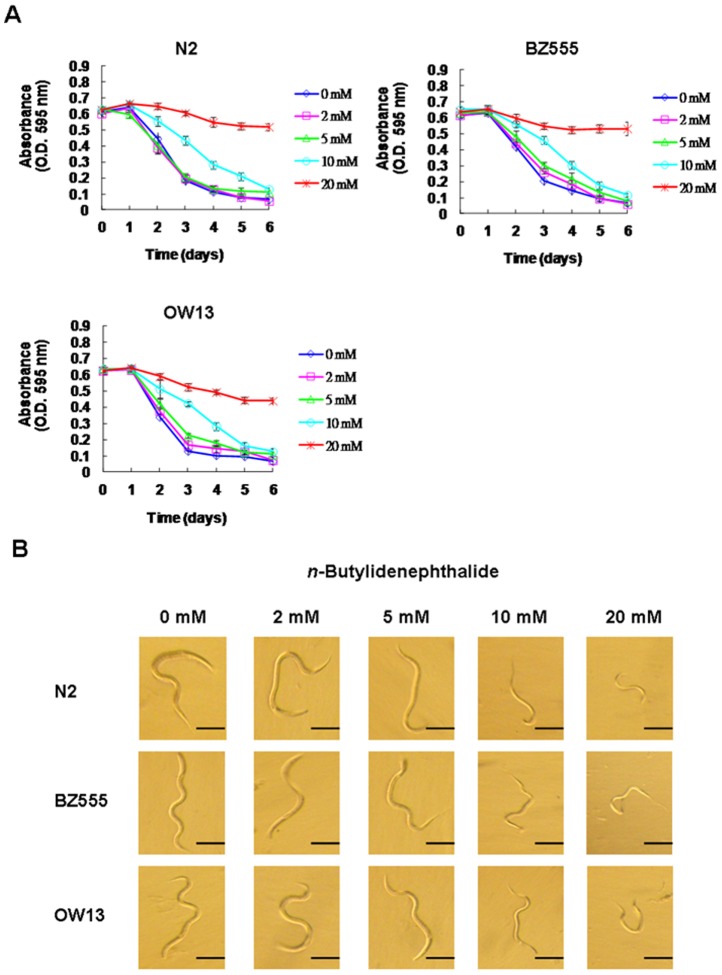
Concentration of *n*-butylidenephthalide for experimenting was determined with a food clearance assay. Between 20 and 30 newly hatched L1 synchronized animals of N2, BZ555 or OW13 were incubated in *E. coli* (OD A_595_ = 0.6) in a 96-well plate at 25°C containing different *n*-butylidenephthalide concentrations to a total volume of 60 µL. The OD of the plate was measured daily for 6 days. Note the lower OD (∼0.6) in a well due to the reduced path length of the 60 µL final suspension compared to the 1 cm path length in a spectrophotometer. (A) The OD of *E. coli* recorded daily for each concentration of *n*-butylidenephthalide. (B) Animals treated with no *n*-butylidenephthalide and the indicated *n*-butylidenephthalide concentrations. Animals treated with 10 mM *n*-butylidenephthalide were alive but concentrations over 20 mM caused death. Scale bar is 100 µm.

To assess the effects of *n*-butylidenephthalide on DA neuron degeneration and α-synuclein accumulation, we first determined the optimal concentrations of *n*-butylidenephthalide to evaluate in our *C. elegans* PD models by food clearance test. Given the advantage of the ability of *C. elegans* to grow in a liquid culture of *E. coli* and the short life cycle, *n*-butylidenephthalide was tested at the rate at which the food source (*E. coli* suspension) was consumed. Each adult animal can generate hundreds of offspring that rapidly consume the limited *E. coli* supply. Therefore, the OD of the wells without *n*-butylidenephthalide significantly reduced in 3 days in N2, BZ555 and OW13 strains (Figure 2A). The DMSO solvent had no influence on the food consumption rate. The addition of 2 mM or 5 mM *n*-butylidenephthalide to the cultures containing N2, BZ555 or OW13 strains showed no effect on food clearance compared to that in control animals, whereas animals treated to 10 mM or 20 mM *n*-butylidenephthalide had significantly delayed food clearance (Figure 2A). Optical observation revealed that animals treated with 10 mM or 20 mM *n*-butylidenephthalide were smaller than untreated animals, whereas animals treated with 2 mM or 5 mM *n*-butylidenephthalide were unaffected (Figure 2B). In addition, animals treated to 20 mM *n*-butylidenephthalide did not generate offspring over the time course of the experiment, which was related with the lack of clearance of the *E. coli* source. This simple assay for determining the concentration range of *n*-butylidenephthalide to test food clearance in *C. elegans* uses a small amount of *n*-butylidenephthalide. In the experiments following this test, animals were exposed with *n*-butylidenephthalide at concentrations of up to 5 mM.

### *n*-Butylidenephthalide attenuated 6-OHDA-induced degeneration of DA neurons

**Figure 3:**
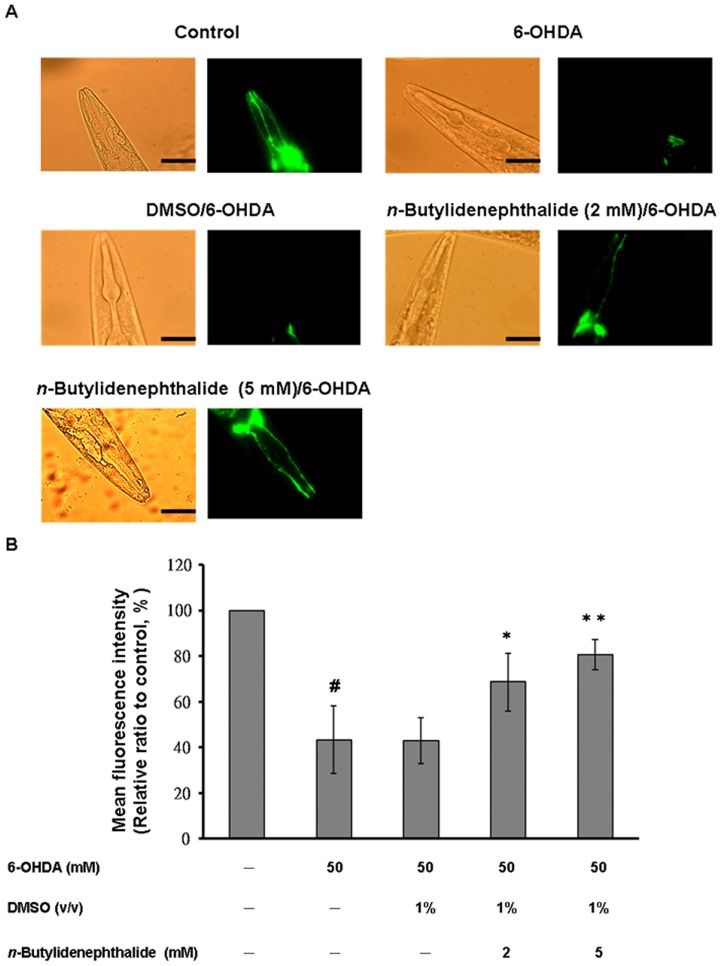
*n*-Butylidenephthalide rescues dopaminergic neurons of *C. elegans* from degeneration resulting from 6-OHDA treatment. (A) GFP expression pattern in dopaminergic neurons of transgenic *C. elegans* strain BZ555. The left side shows the differential interference contrast (DIC) image. The right side shows the fluorescence images. Scale bar, 50 µm. (B) Graphical representation for fluorescence intensity of GFP expression pattern in dopaminergic neurons of a transgenic *C. elegans* strain BZ555 as quantified using AxioVision software. The data represent the mean ± SD (n = 10). A hash (#) indicates significant differences between 6-OHDA-treated and untreated animals (*p*<0.01); an asterisk (*) indicates significant differences between the 6-OHDA-treated control samples and the *n*-butylidenephthalide/6-OHDA-treated samples (^*^ *p*<0.05, ^**^ *p*<0.01).

*C. elegans* contains accurately eight DA neurons, including one pair of anterior deirid (ADE) neurons and two pairs of cephalic (CEP) neurons in the head position, and one pair of posterior deirid (PDE) neurons in the posterior lateral region. Selective degeneration of these DA neurons was observed through treatment to 6-OHDA. To analyze *n*-butylidenephthalide efficacy, we assessed neuronal viability by measuring the loss of expression of a GFP reporter gene in DA neurons of 6-OHDA-treated BZ555 animals. We found that CEP and ADE neurons showed a partial GFP loss with slight reduction in GFP expression in PDE neurons after 6-OHDA treatment (Figure 3A). The DMSO solvent had no influence on 6-OHDA-induced DA neuron degeneration. When animals were exposed with *n*-butylidenephthalide, remarkable protection was noticed in DA neurons with CEP, and ADE neurons presenting an augmented expression of GFP (Figure 3A). We further measured the fluorescence intensity in DA neurons using AxioVision software. In 6-OHDA-treated animals, the mean fluorescence (GFP) intensity reduced by about 57% (*p*<0.01) compared to that of untreated animals (Figure 3B). *n*-Butylidenephthalide increased the GFP expression in a dose-dependent manner. At 5 mM *n*-butylidenephthalide, the fluorescence intensity of GFP expression in DA neurons of 6-OHDA-treated animals raised by about 1.9-fold (*p*<0.01) compared to that in animals treated only with 6-OHDA (Figure 3B).

### *n*-butylidenephthalide arrested accumulation of α-synuclein protein

**Figure 4:**
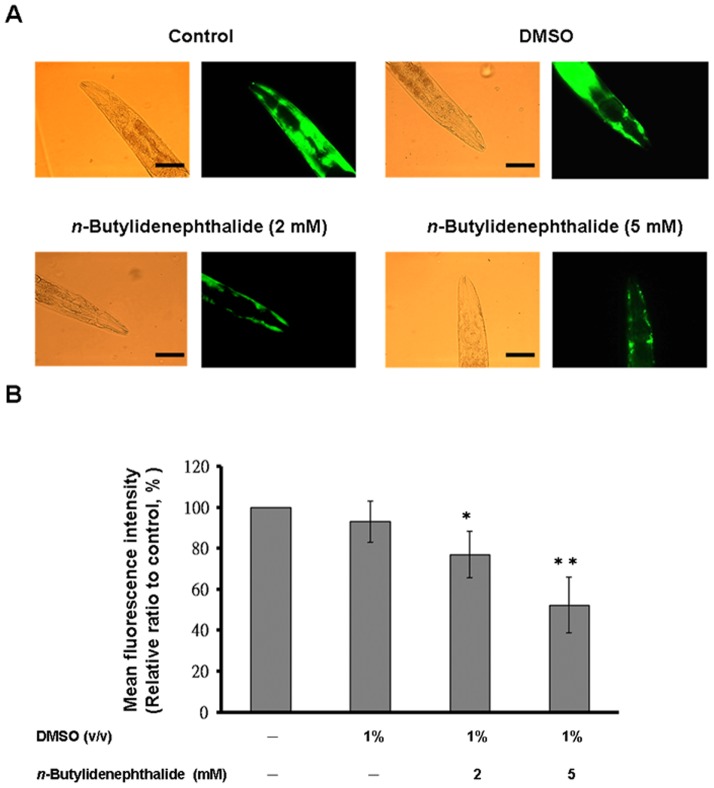
*n*-Butylidenephthalide reduces α-synuclein accumulation in the OW13 strain of *C. elegans*. (A) YFP expression pattern in muscles of transgenic *C. elegans* strain OW13. The left side shows the differential interference contrast (DIC) image. The right side shows fluorescence images. Scale bar, 50 µm. (B) Graphical representation for fluorescence intensity of YFP expression pattern in muscles of transgenic *C. elegans* strain OW13 as quantified using AxioVision software. The data represent the mean ± SD (n = 10). An asterisk (*) indicates significant differences between the control samples and the *n*-butylidenephthalide-treated samples (^*^ *p*<0.05, ^**^ *p*<0.01).

*C. elegans* does not contain an orthologous gene of α-synuclein. However, the genetic flexibility of the nematode allows transgenic expression of human α-synuclein genes and the investigation of α-synuclein accumulation. OW13 animals of untreated and *n*-butylidenephthalide*-*treated groups were measured for their α-synuclein protein accumulation. The DMSO solvent had no influence on α-synuclein accumulation. Treatment of animals with *n*-butylidenephthalide showed significantly reduced fluorescence intensity of accumulation compared to that of untreated animals (Figure 4A). *n*-butylidenephthalide decreased the YFP expression of OW13 animals in a dose-dependent manner. At 5 mM *n*-butylidenephthalide, the fluorescence intensity of YFP expression associated with α-synuclein protein accumulation in OW13 animals lessened by about 48% (*p*<0.01) compared to that in untreated animals (Figure 4B).

### *n*-butylidenephthalide restored lipid content in a transgenic C. elegans model overexpressing human α-synuclein

**Figure 5:**
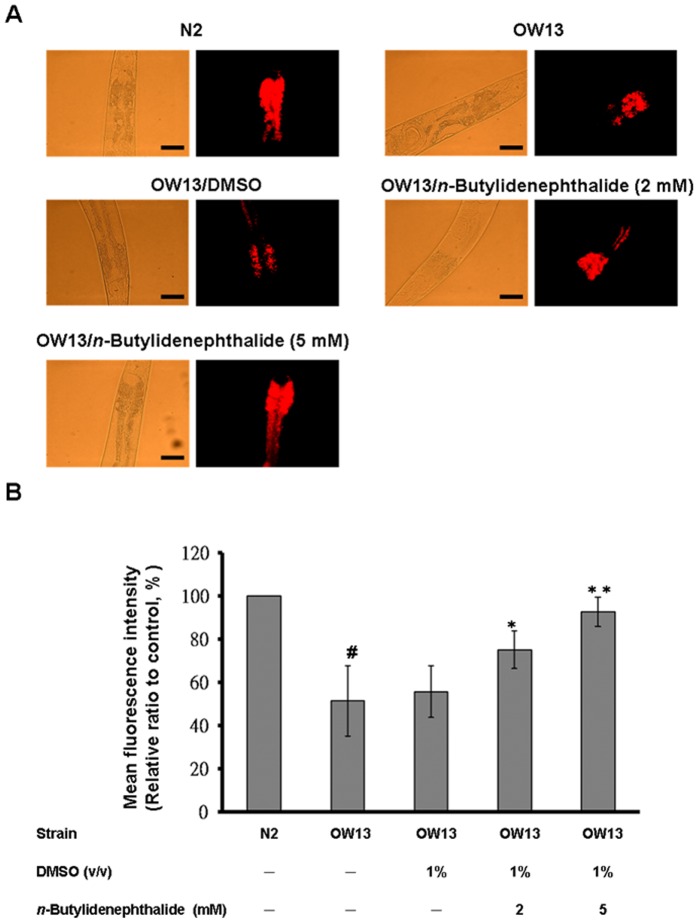
*n*-Butylidenephthalide elevates lipid content in the OW13 strain of *C. elegans*. (A) Nile red staining pattern in transgenic *C. elegans* strain OW13. The left side shows the differential interference contrast (DIC) image. The right side shows fluorescence images. Scale bar, 50 µm. (B) Graphical representation for fluorescence intensity of the Nile red pattern in transgenic *C. elegans* strain OW13 as quantified using AxioVision software. The data represent the mean ± SD (n = 10). A hash (#) indicates significant differences between N2 and OW13 animals (*p*<0.01); an asterisk (*) indicates significant differences between the OW13 control samples and the *n*-butylidenephthalide-treated samples (^*^ *p*<0.05, ^**^ *p*<0.01).

PD is reported to be connected with altered levels of fatty acids and lipid content by α-synuclein expression [62]. We assayed the lipid levels in OW13 animals untreated or treated with *n*-butylidenephthalide. Nile red staining was employed to fluorescently label the lipids within animals. Animals of the control group N2 revealed an optimum level of lipids. The lipid content in α-synuclein-overexpressing OW13 animals was reduced. The DMSO solvent had no influence on the lipid level (Figure 5A). *n*-Butylidenephthalide raised the lipid content of OW13 animals in a dose-dependent manner. At 5 mM *n*-butylidenephthalide treatment, the fluorescence intensity of Nile red in the entire body of OW13 animals augmented by about 1.8-fold (*p*<0.01) compared to that in untreated OW13 animals (Figure 5B).

### *n*-Butylidenephthalide recovered food-sensing behavior of 6-OHDA-exposed *C. elegans*

**Figure 6:**
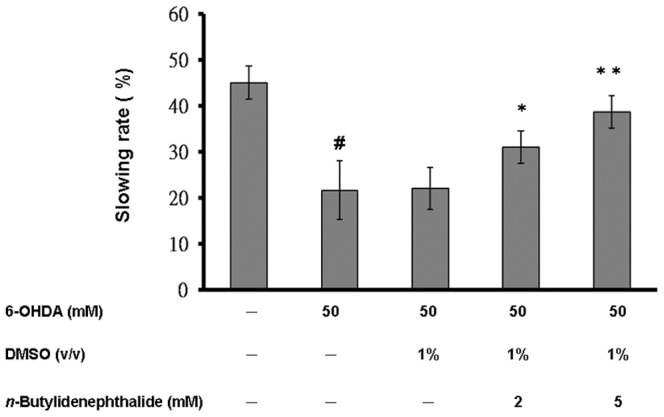
*n*-Butylidenephthalide improves food-sensing behavior in 6-OHDA-treated N2 *C. elegans*. The locomotory rate (frequency of bending) of 6-OHDA-untreated animals, 6-OHDA-treated animals, or *n*-butylidenephthalide/6-OHDA-treated animals with or without bacteria lawns was assayed. Shown are the slowing rates calculated as the percentage decrease of the locomotory rate in the bacteria lawn as compared with that in no bacteria lawn. The data represent the mean ± SD (n = 10). A hash (#) indicates significant differences between 6-OHDA-treated and untreated animals (*p*<0.001); an asterisk (*) indicates significant differences between the 6-OHDA-treated control samples and the *n*-butylidenephthalide/6-OHDA-treated samples (*p<0.05, **p<0.01).

It has been confirmed that 6-OHDA-exposed animals show a phenotype of DA neuron degeneration that should affect dopamine synthesis and food-sensing behavior [45], [57]. *C. elegans* move themselves by bending their bodies for transportation, and the rate of movement is estimated by the bending frequency. Once animals come across food, they lessen the bending frequency to feed themselves more effectively. 6-OHDA-treated animals, however, lose to display a decremental bending frequency in response to food sensing. Accordingly, the function of dopamine neurotransmission in *C. elegans* is linked with this food-sensing behavior. We examined whether 6-OHDA treatment in *C. elegans* generates a defect in this function in 3-day-old animals that were synchronized for age. Wild-type N2 animals revealed a 45% lessening in bending frequency upon contact with bacteria (Figure 6). In contrast, 6-OHDA-treated animals showed a significant decreasing in this decremental response compared with wild-type N2 animals (22%, *p*<0.01). The DMSO solvent had no influence on the 6-OHDA-induced lessening in the decremental response. *n*-butylidenephthalide recovered the decremental response of 6-OHDA-treated animals in a dose-dependent manner. At 5 mM *n*-butylidenephthalide treatment, 6-OHDA-treated animals displayed reduced bending movement upon contact with bacteria by about 1.8-fold (*p*<0.01) compared with that in untreated animals (Figure 6).

### *n*-butylidenephthalide restored the level of dopamine of 6-OHDA-treated *C. elegans*

**Figure 7:**
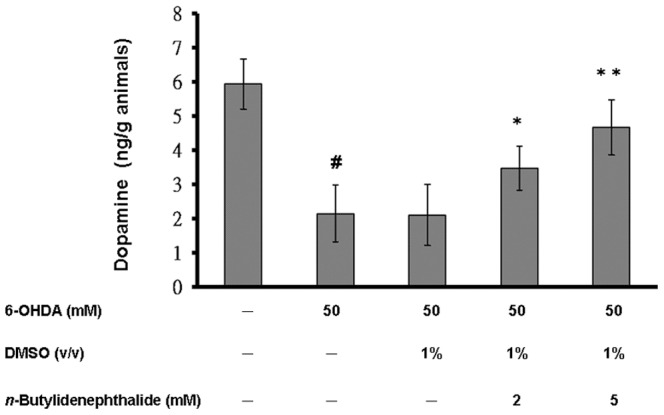
*n*-Butylidenephthalide raises DA content in 6-OHDA-treated N2 *C. elegans*. Shown is quantitation of DA levels (ng/g wet weight of animals) by HPLC-chemiluminescence detection in 6-OHDA-untreated animals, 6-OHDA-treated animals, or *n*-butylidenephthalide/6-OHDA-treated animals. Three-day-old animals synchronized for age were used. The data represent the mean ± SD (n = 3). A hash (#) indicates significant differences between 6-OHDA-treated and untreated animals (*p*<0.001); an asterisk (*) indicates significant differences between the 6-OHDA-treated control samples and the *n*-butylidenephthalide/6-OHDA-treated samples (*p<0.05, **p<0.01).

We next measured the level of dopamine in 3-day-old wild-type N2, 6-OHDA-treated, or *n*-butylidenephthalide/6-OHDA-treated animals that were synchronized for age. N2 animals comprised ∼6 ng of dopamine per gram of animals. In 6-OHDA-treated animals, the level of dopamine reduced by about 64% (*p*<0.01) compared to that of untreated animals (Figure 7). The DMSO solvent had no influence on 6-OHDA-induced lessening in the level of dopamine. *n*-butylidenephthalide augmented the level of dopamine in a dose-dependent manner. At 5 mM *n*-butylidenephthalide, the level of dopamine of 6-OHDA-treated animals raised by about 2.2-fold (*p*<0.01) compared to that in animals treated only with 6-OHDA (Figure 7).

### *n*-butylidenephthalide prolonged the life span of 6-OHDA-treated animals

**Figure 8:**
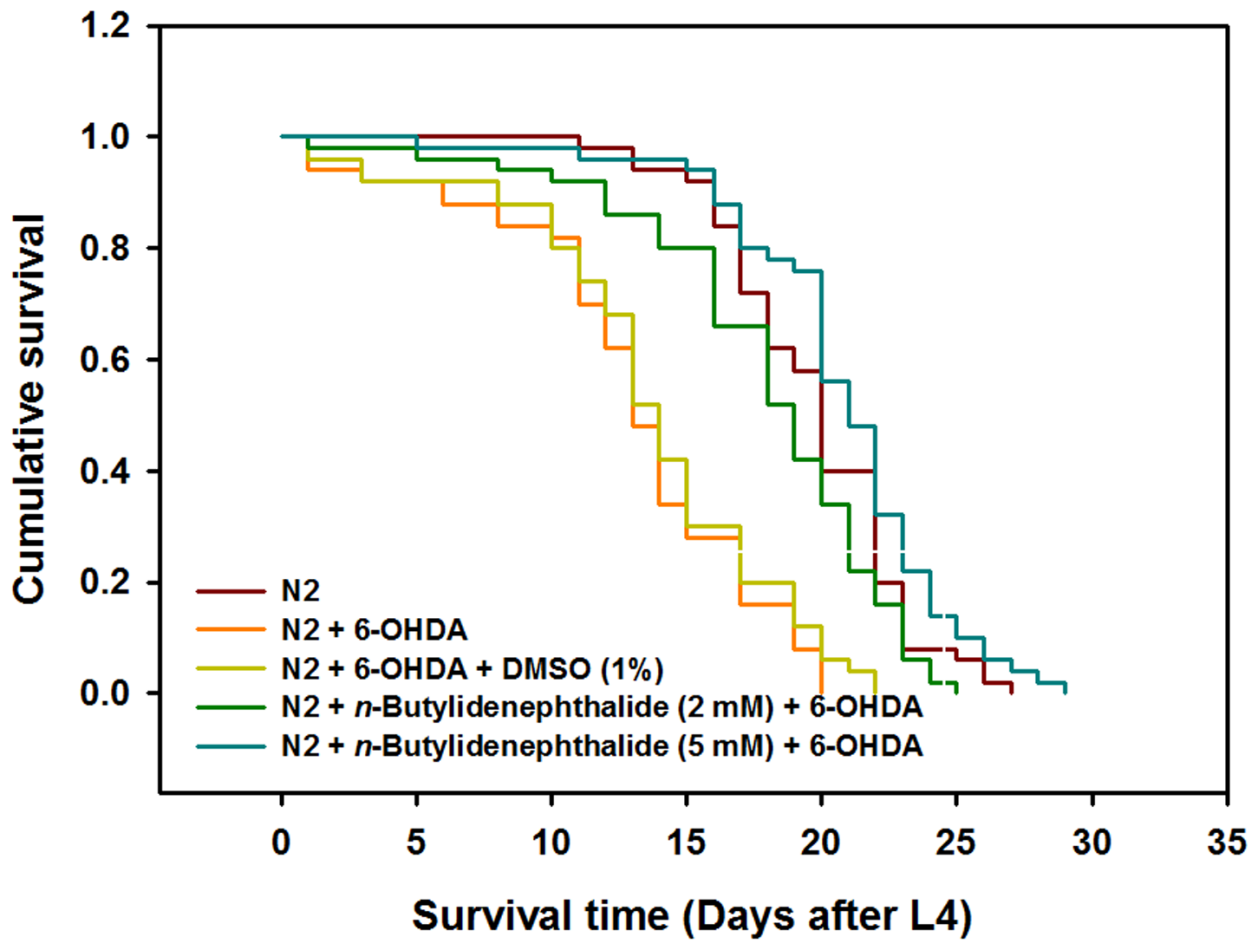
*n*-Butylidenephthalide prolongs longevity in 6-OHDA-treated N2 *C. elegans*. Cumulative survival curves of wild-type N2 animals grown on OP50, 6-OHDA-treated animals grown on OP50, and *n*-butylidenephthalide/6-OHDA-treated animals fed on *n*-butylidenephthalide/OP50.

The effect of *n*-butylidenephthalide on the longevity of 6-OHDA-treated animals was observed. 6-OHDA-exposed animals have a shorter life span compared to wild-type N2 animals (Figure 8). The DMSO solvent had no influence on the longevity of 6-OHDA-treated animals. *n*-butylidenephthalide increased the life span in a dose-dependent manner. We noted that 5 mM of *n*-butylidenephthalide appreciably improved the life span of 6-OHDA-treated animals. Figure 8 represents the cumulative survival patterns, as calculated by Kaplan–Meier survival analysis of each group. The mean survival for the *n*-butylidenephthalide/6- OHDA (5 mM) group was 21.82±2.11 days vs. 13.00±2.43 days for the 6-OHDA condition (*p*<0.01).

### *n*-butylidenephthalide lessened aopotosis modulator *egl-1* expression in 6-OHDA-exposed *C. elegans*

**Figure 9:**
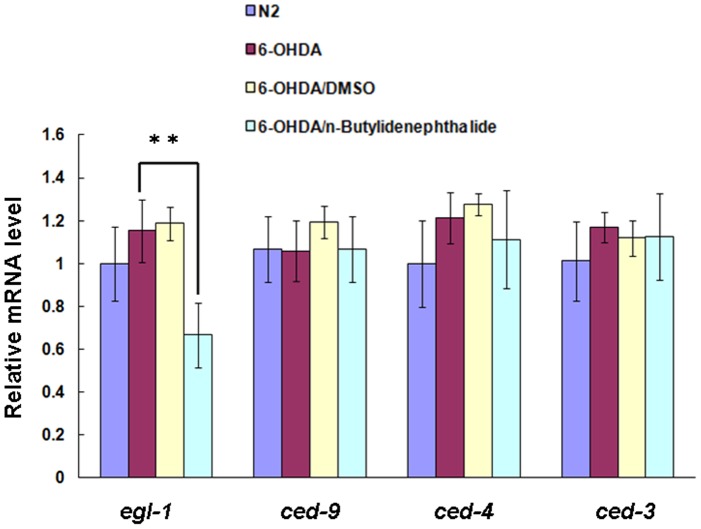
*n*-Butylidenephthalide lessens *egl-1* expression in apoptosis modulation in 6-OHDA-treated *C. elegans*. Quantitative real-time RT-PCR experiments show the expression levels of *egl-1*, *ced-3*, *ced-4* and *ced-9* using RNAs isolated from N2, 6-OHDA-treated, or *n*-butylidenephthalide/6-OHDA-treated animals. The data represent the mean ± SD (n = 3). An asterisk (*) indicates significant differences between the 6-OHDA-treated control samples and the *n*-butylidenephthalide/6-OHDA-treated samples (**p<0.01).

We hypothesized that a key aspect of the apoptosis pathway might be regulated in the 6-OHDA-treated animals by *n*-butylidenephthalide. To evaluate whether the observed attenuation in DA neuron degeneration of *C. elegans* was the result of lessened apoptosis activity, after treating the animals with *n*-butylidenephthalide, we employed a real-time PCR technique to determine the mRNA levels of *egl-1*, *ced-3*, *ced-4* and *ced-9*, which are associated with apoptosis of *C. elegans*. As represented in Figure 9, the expression level of *egl-1*, *ced-3*, *ced-4* and *ced-9* was not raised in 6-OHDA-treated animals compared to that in untreated animals. The DMSO solvent had no influence on the expression level of *egl-1*, *ced-3*, *ced-4* and *ced-9* in 6-OHDA-treated animals. At 5 mM *n*-butylidenephthalide, the expression level of *egl-1* in 6-OHDA-treated animals reduced by about 42% (*p*<0.01) compared to that in animals treated only with 6-OHDA (Figure 9).

### *n*-butylidenephthalide enhanced somatic proteasome activity by raising proteasome regulatory subunit *rpn-6* expression in a transgenic C. elegans model overexpressing human α-synuclein

**Figure 10:**
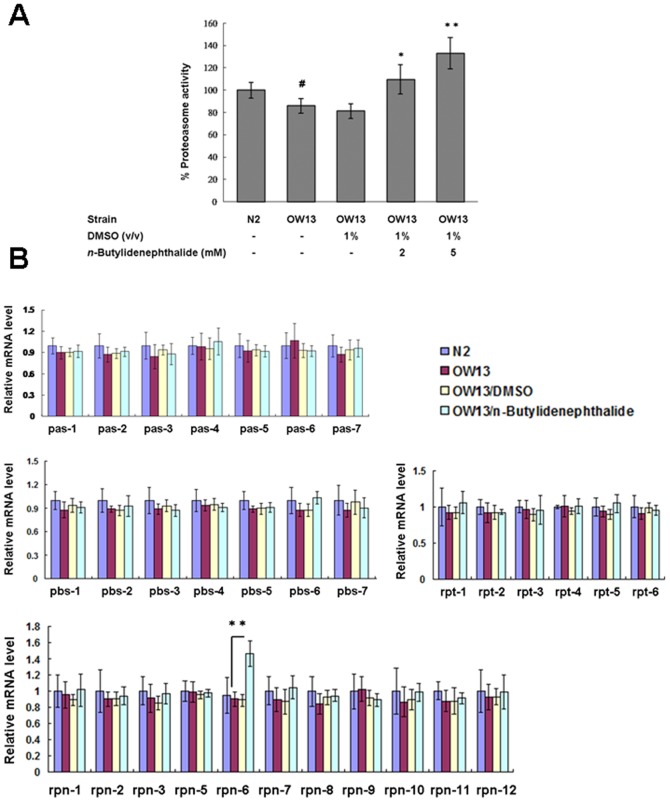
*n*-Butylidenephthalide augments proteasome activity by enhancing the expression of *rpn-6* in the OW13 strain of *C. elegans*. (A) *n*-Butylidenephthalide augments proteasome activity in the OW13 strain of *C. elegans*. Chymotrypsin-like activity of the proteasome was monitored by Z-Gly-Gly-Leu-AMC digestion in a day 3 adult animal extract containing equal amounts of total protein. The data represent the mean ± SD (n = 3). A hash (#) indicates significant differences between N2 and OW13 animals (*p*<0.05); an asterisk (*) indicates significant differences between the OW13 control samples and the *n*-butylidenephthalide-treated OW13 samples (*p<0.05, **p<0.01). (B) *n*-Butylidenephthalide enhances the expression of *rpn-6* of proteasome in the OW13 strain of *C. elegans*. Quantitative real-time RT-PCR experiments show the expression level of individual subunit of the 26S proteasome, using RNAs isolated from OW13 control or *n*-butylidenephthalide-treated animals. The data represent the mean ± SD (n = 3). An asterisk (*) indicates significant differences between the OW13 control samples and the *n*-butylidenephthalide-treated samples (**p<0.01).

We hypothesized that a key aspect of the proteostasis network, the ubiquitin proteasome system, might be regulated in *n*-butylidenephthalide-treated OW13 animals. To evaluate whether the observed diminishing in α-synuclein accumulation in the muscle of OW13 animals was the result of elevated proteasomal activity, we analyzed 26S proteasome activity upon treatment with *n*-butylidenephthalide by employing a proteasome activity assay with a fluorescent substrate. As represented in Figure 10A, the level of chymotrypsin-like proteasome activity was about 14% lower in OW13 animals compared to that in N2 animals (*p*<0.05). The DMSO solvent had no influence on the proteasome activity of OW13 animals. *n*-Butylidenephthalide treatment significantly raised the chymotrypsin-like proteasome activity in OW13 animals in a dose-dependent manner. Chymotrypsin-like proteasome activity following 5 mM *n*-butylidenephthalide treatment was augmented by about 1.5-fold in the OW13 animals (*p*<0.01) (Figure 10A). These results indicate that elevated proteasome activity results in a reduction in α-synuclein accumulation and that *n*-butylidenephthalide treatment can increase proteasome activity in the animal model of PD. We therefore next assessed whether the proteasome activity of *n*-butylidenephthalide-treated OW13 animals linked with a raised expression level of the catalytically active subunits of the 20S proteasome or the regulatory particles of the 19S proteasome. The level of all subunits was not different in OW13 animals compared to that in N2 animals. The DMSO solvent had no influence on the subunit expression of OW13 animals. *n*-Butylidenephthalide raised the expression level of the *rpn-6* of regulatory subunit. The expression level of *rpn-6* following 5 mM *n*-butylidenephthalide treatment was enhanced by about 1.6-fold in the OW13 animals (*p*<0.01) (Figure 10B).

## Discussion

In this research, we established strategies for assessing the therapeutic efficacy of phytocompounds in *C. elegans* PD models. Our methods use the advantages of the *C. elegans* model for drug testing, including easy and exact visualization of live DA neurons and α-synuclein accumulation. Moreover, small-scale liquid cultures significantly decrease the amount of the drug required for examination, and molecular biology methods indicate specific cellular pathways in the activity of the compound. These assays could be powerful for inexpensive, rapid examination and screening of large numbers of drugs for PD. Our data show that *n*-butylidenephthalide reduces DA neuron degeneration; attenuates α-synuclein accumulation; arrests lipid loss; and restores food-sensing behavior and the level of dopamine in a pharmacological or transgenic *C. elegans* model. Furthermore, *n*-butylidenephthalide raises the life span of 6-OHDA-treated animals. To the best of our knowledge, this is the first report of the antiparkinsonian ability of *n*-butylidenephthalide in an animal model.

Given that DA neurons are particularly sensitive to oxidative stress, reactive oxygen species are major regulators in neuronal cell apoptosis and death. 6-OHDA impairs neurons by producing reactive oxygen species such as the superoxide radical [63]. Recently, it was demonstrated that *n*-Butylidenephtaline possesses anti-oxidant activities. The transcription factor Nrf2 mediates the expression of many cellular anti-oxidative stress genes. Data showed that *n*-Butylidenephtaline activates the Nrf2 pathway, and then protects against oxidative stress [64]. Therefore, the neuroprotective role of *n*-butylidenephthalide in 6-OHDA-induced DA neuron lesion, food-sensing behavior defects, dopamine loss, and longevity shortening is possibly associated with its antioxidant activity. It has also been shown that mitochondrial damage induced by 6-OHDA causes release of cytochrome c to cytosol and the activation of caspase-3 [65]. Caspase-3 is an important effector in apoptosis that is induced via different pathways in various mammalian cell types, mainly in the cytochrome c-dependent apoptosis pathways [66]. In *C. elegans*, the BH3-only domain protein EGL-1, the Apaf-1 homolog CED-4 and the CED-3 caspase participate apoptosis induction, whereas the Bcl-2 homolog CED-9 arrests apoptosis. CED-9 is thought to inhibit CED-4 by blocking CED-4 accumulation in the perinuclear space in response to proapoptotic stimuli. EGL-1 antagonizes CED-9, leading to CED-4 oligomerization and triggering apoptosis through CED-3 caspase activation [67], [68]. Nuclei of DA neuron of 6-OHDA-exposed *C. elegans* also revealed dark and chromatin condensation, characteristics that are consistent with apoptotic cells [57]. Therefore, in this study, we demonstrated that *n*-butylidenephthalide lessened the expression of *egl-1*, which, in turn, may counter 6-OHDA-induced DA neuron apoptosis. The detailed mechanism requires further investigation.

To observe accumulation of α-synuclein, we used the C. elegans strain OW13. It was overexpressed human α-synuclein fused to yellow fluorescent protein under control of the unc-54 promoter, which directs expression to the body wall muscle cells [69]. Muscle expression rather than neuronal expression was decided for three reasons. First, the unc-54 promoter is strong and muscle cells are the largest, most easily scored cell type, allowing for exact measurement of α-synuclein accumulation and its subcellular localization. Second, muscle expression has been employed successfully to determine modifier genes of PD in previous studies [33], [36], [49], . Third, the accumulation of α-synuclein in the muscle resulted in PD-like progressive decline of motility in *C. elegans*, indicating the in vivo toxicity of these aggregates [40], [73]. In our study, we implied that the anti-accumulation effect of *n*-butylidenephthalide may associate with its mediation of the proteosome system. It is well known that ubiquitin-proteosome system promotes cell survival from harm under environmental stressful conditions [74]. The 26S proteasome complex of *C. elegans* comprises a 20S "catalytic core", a huge protein complex that harbors the proteolytically active centres and 19S "regulatory caps", which are responsible for recognition of poly-ubiquitinated protein substrates targeted for degradation. 19S can be further divided into two distinct substructures including a ring of six homologous ATPase subunits of the AAA family (Rpt-1–6), eight essential RP non-ATPase subunits (Rpn-3, Rpn-5–9 and Rpn-11–12), two α-helical solenoid structures (Rpn-1 and Rpn-2), and two Ub receptors (Rpn-10) [75]. The effect of *n*-butylidenephthalide may be linked with enhanced expression of *rpn-6*, a subunit of the 19S proteasome. Rpn-6 is a candidate to correct deficiencies in age-related protein homeostasis disorders. Ectopic expression of *rpn-6* is sufficient to confer proteotoxic stress resistance and extend lifespan [61]. The exact mechanisms underlying these results will require further investigation.

The abundance of lipid molecules in the central nervous system suggests that their function is not restricted to the structural components and energy of cells. Some lipids in the central nervous system are well-known to play a key role in neurotransmission. Disorders in cellular signaling have been connected to almost every neurodegenerative disease [76]. Spatial and temporal aspects of cellular signaling activities are in part modulated by lipid components that can change protein location and scaffolding events through a dynamic control of membrane microdomains [77]. This protective effect of *n*-butylidenephthalide could lessen α-synuclein accumulation, therefore decreasing lipid peroxidation. Hence, such a protective effect may regulate the disturbed arrangement of lipids and thus maintains efficient cellular signaling.

Evidence suggests that chronic neuroinflammation is linked with the pathophysiology of PD [78]. Activation of microglia and raised levels of pro-inflammatory mediators such as TNF-α, IL-1β and IL-6 have been confirmed in the substantia nigra of PD patients and in animal models of PD [79]. It is hypothesized that activated microglia secrete high levels of proinflammatory mediators that impair neurons and further activate microglia, leading to further promoting of inflammation and neurodegeneration. Moreover, DA neurons are more sensitive to proinflammatory mediators than other cell types are [80]. In our previous study, *n*-butylidenephthalide attenuated the maturation of mouse dendritic cells via blockage of IκB kinase/nuclear factor-κB activities [17]. Therefore, *n*-butylidenephthalide may also be able to modulate inflammation in the brains of patients with PD and lessen DA neuron damage.

Some reports have implied that phytocompounds, including acacetin [81], curcumin [82], gastrodin [83], isoliquiritigenin [65] and quercetin [84], have special neuroprotective effects on DA neurons. In addition, isorhynchophylline promotes the degradation of α-synuclein in neuronal cells via inducing autophagy [85]. The current study indicates that *n*-butylidenephthalide is a new member on the list of phytocompounds with these effects. Natural antioxidant phytocompound curcumin showed neuroprotective capacity in the 6-OHDA model of PD [86], [87] and also attenuated aggregation of α-synuclein in cell model of PD [88], [89]. The simpler anti-oxidant N-acetylcysteine prevents loss of DA neurons in the EAAC1-/- mouse [90]. Oral N-acetylcysteine reduced loss of dopaminergic terminals in α-synuclein overexpressing mice model [91]. Vitamin E also exhibited beneficial effects in PD [92]. Our study indicates that *n*-butylidenephthalide, although less studied, possesses several pharmacological properties that cover with those of the more broadly investigated curcumin, N-acetylcysteine, and vitamin E in PD. Moreover, according our supplement data, *n*-butylidenephthalide showed neuroprotective capacity in the 6-OHDA-treated BZ555 animals as well as curcumin and N-acetylcysteine, and better than vitamin E (Figure S1). Furthermore, *n*-butylidenephthalide revealed the power of anti-accumulation in the OW13 animals better than curcumin, N-acetylcysteine, and vitamin E (Figure S2). Therefore, *n*-butylidenephthalide has better potential in pharmaceutics for PD. The available data on the potential clinical applications of *n*-butylidenephthalide are increasing. The *in vivo* influences of *n*-butylidenephthalide treatment have been shown in a rat model [16], [93]. Further clinical trials are required to assess the suitability of *n*-butylidenephthalide for disease control.

These results support traditional Chinese medicine practitioners with information about the use of herbs containing *n*-butylidenephthalide in the treatment of neuron-related disorders. This readily available agent allows a low-cost, convenient, and highly effective means of modifying the survival and function of DA neurons. In the future, we will address the exact mechanism by which *n*-butylidenephthalide maintains DA neuron activity in other animal PD models, which may develop the novel antiparkinson potential of *n*-butylidenephthalide in the prevention and treatment of PD.

Recent reports have shown that incorporation of phytocompounds into liposome or nanoparticles improves in the oral delivery of a drug [94]–[96], as these particles can avoid it degradation in the gastrointestinal tract; as a result of their individual absorption mechanism through the circulation system, these particles also shield the drug from the first-pass effect in the liver and allow continued release at the correct site of action. Such techniques will be exploited for improvements in the clinical applications of *n*-butylidenephthalide.

## Supporting Information

Figure S1 **The neuroprotective effects of** ***n*** **-butylidenephthalide, curcumin, N-acetylcysteine and vitamin E on 6-OHDA-induced degeneration of DA neurons in** ***C. elegans.*** Curcumin, N-acetylcysteine and vitamin E were purchased from Sigma-Aldrich (St. Louis, MO). The addition of 5 mM curcumin, N-acetylcysteine and vitamin E individual to the cultures containing transgenic *C. elegans* strain BZ555 revealed no effect on food clearance assay compared to that in control animals (data not shown). Graphical representation for fluorescence intensity of GFP expression pattern in DA neurons of transgenic *C. elegans* strain BZ555 as quantified using AxioVision software. The data represent the mean ± SD (n = 10). A hash (#) indicates significant differences between 6-OHDA-treated and untreated animals (*p*<0.001); an asterisk (*) indicates significant differences between the 6-OHDA-treated control samples and the *n*-butylidenephthalide, curcumin, N-acetylcysteine or vitamin E/6-OHDA-treated samples (^*^ *p*<0.05, ^**^ *p*<0.01).
(DOC)

Figure S2 **The anti-accumulation effects of** ***n*** **-butylidenephthalide, curcumin, N-acetylcysteine and vitamin E in the OW13 strain of** ***C. elegans*** **.** Curcumin, N-acetylcysteine and vitamin E were purchased from Sigma-Aldrich (St. Louis, MO). The addition of 5 mM curcumin, N-acetylcysteine and vitamin E individual to the cultures containing transgenic *C. elegans* strain OW13 revealed no effect on food clearance assay compared to that in control animals (data not shown). Graphical representation for fluorescence intensity of YFP expression pattern in muscles of transgenic *C. elegans* strain OW13 as quantified using AxioVision software. The data represent the mean ± SD (n = 10). An asterisk (*) indicates significant differences between the control samples and the *n*-butylidenephthalide, curcumin, N-acetylcysteine or vitamin E-treated samples (^*^ *p*<0.05, ^**^ *p*<0.01).
(DOC)

Table S1 **List of primers used for qPCR assays.**
(DOC)
