# Supplementary material for: n-Butylidenephthalide Protects against Dopaminergic Neuron Degeneration and α-Synuclein Accumulation in Caenorhabditis elegans Models of Parkinson's Disease
Source: PLoS One. 2014 Jan 8;9(1):e85305. doi: 10.1371/journal.pone.0085305 (PMC3885701; doi:10.1371/journal.pone.0085305)
Supplement: Figure S1 — The neuroprotective effects of n -butylidenephthalide, curcumin, N-acetylcysteine and vitamin E on 6-OHDA-induced degeneration of DA neurons in C. elegans. Curcumin, N-acetylcysteine and vitamin E were purchased from Sigma-Aldrich (St. Louis, MO). The addition of 5 mM curcumin, N-acetylcysteine and vitamin E individual to the cultures containing transgenic C. elegans strain BZ555 revealed no effect on food clearance assay compared to that in control animals (data not shown). Graphical representation for fluorescence intensity of GFP expression pattern in DA neurons of transgenic C. elegans strain BZ555 as quantified using AxioVision software. The data represent the mean ± SD (n = 10). A hash (#) indicates significant differences between 6-OHDA-treated and untreated animals (p<0.001); an asterisk (*) indicates significant differences between the 6-OHDA-treated control samples and the n-butylidenephthalide, curcumin, N-acetylcysteine or vitamin E/6-OHDA-treated samples (* p<0.05, ** p<0.01). (DOC) [file pone.0085305.s001.doc]

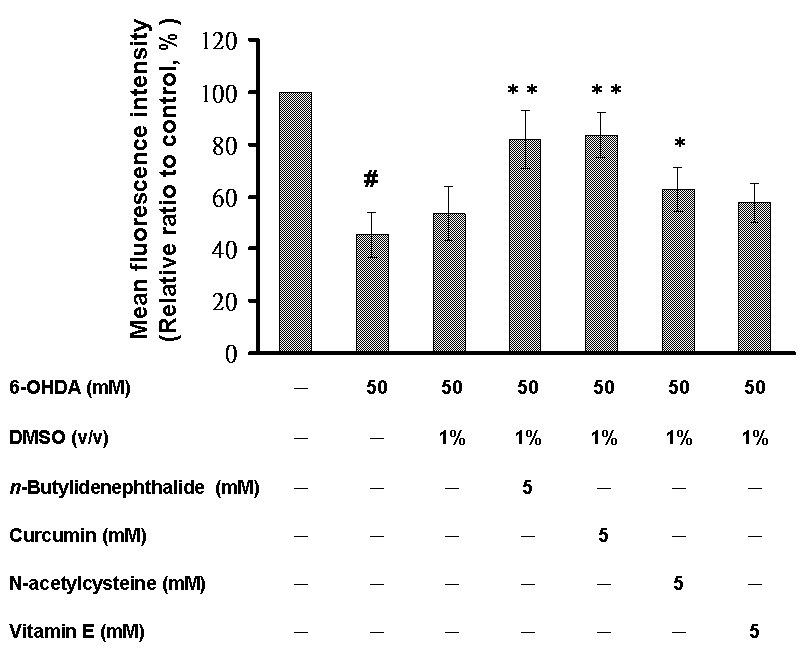


**Figure S1. The neuroprotective effects of *n*-butylidenephthalide, curcumin, N-acetylcysteine and vitamin E on 6-OHDA-induced degeneration of DA neurons in *C. elegans.*** Curcumin, N-acetylcysteine and vitamin E were purchased from Sigma-Aldrich (St. Louis, MO). The addition of 5 mM curcumin, N-acetylcysteine and vitamin E individual to the cultures containing transgenic *C. elegans* strain BZ555 revealed no effect on food clearance assay compared to that in control animals (data not shown). Graphical representation for fluorescence intensity of GFP expression pattern in DA neurons of transgenic *C. elegans* strain BZ555 as quantified using AxioVision software. The data represent the mean ± SD (n = 10). A hash (#) indicates significant differences between 6-OHDA-treated and untreated animals (*p* < 0.001); an asterisk (*) indicates significant differences between the 6-OHDA-treated control samples and the *n*-butylidenephthalide, curcumin, N-acetylcysteine or vitamin E/6-OHDA-treated samples (^*^*p* < 0.05, ^**^*p* < 0.01).
