# Supplementary material for: n-Butylidenephthalide Protects against Dopaminergic Neuron Degeneration and α-Synuclein Accumulation in Caenorhabditis elegans Models of Parkinson's Disease
Source: PLoS One. 2014 Jan 8;9(1):e85305. doi: 10.1371/journal.pone.0085305 (PMC3885701; doi:10.1371/journal.pone.0085305)
Supplement: Figure S2 — The anti-accumulation effects of n -butylidenephthalide, curcumin, N-acetylcysteine and vitamin E in the OW13 strain of C. elegans . Curcumin, N-acetylcysteine and vitamin E were purchased from Sigma-Aldrich (St. Louis, MO). The addition of 5 mM curcumin, N-acetylcysteine and vitamin E individual to the cultures containing transgenic C. elegans strain OW13 revealed no effect on food clearance assay compared to that in control animals (data not shown). Graphical representation for fluorescence intensity of YFP expression pattern in muscles of transgenic C. elegans strain OW13 as quantified using AxioVision software. The data represent the mean ± SD (n = 10). An asterisk (*) indicates significant differences between the control samples and the n-butylidenephthalide, curcumin, N-acetylcysteine or vitamin E-treated samples (* p<0.05, ** p<0.01). (DOC) [file pone.0085305.s002.doc]

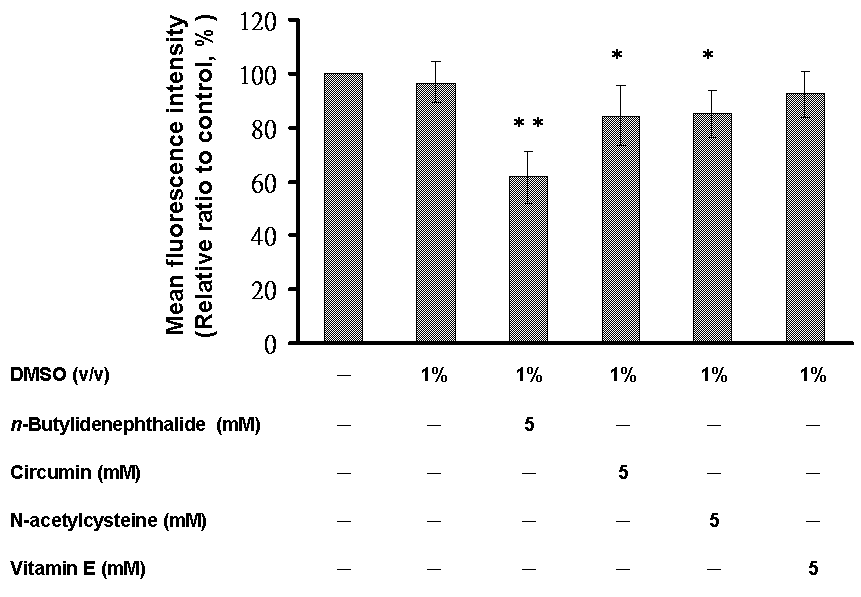


**Figure S2. The anti-aggregates effects of *n*-butylidenephthalide, curcumin, N-acetylcysteine and vitamin E in the OW13 strain of *C. elegans*.** Curcumin, N-acetylcysteine and vitamin E were purchased from Sigma-Aldrich (St. Louis, MO). The addition of 5 mM curcumin, N-acetylcysteine and vitamin E individual to the cultures containing transgenic *C. elegans* strain OW13 revealed no effect on food clearance assay compared to that in control animals (data not shown). Graphical representation for fluorescence intensity of YFP expression pattern in muscles of transgenic *C. elegans* strain OW13 as quantified using AxioVision software. The data represent the mean ± SD (n = 10). An asterisk (*) indicates significant differences between the control samples and the *n*-butylidenephthalide, curcumin, N-acetylcysteine or vitamin E-treated samples (^*^*p* < 0.05, ^**^*p* < 0.01).
