# Supplementary material for: n-Butylidenephthalide Protects against Dopaminergic Neuron Degeneration and α-Synuclein Accumulation in Caenorhabditis elegans Models of Parkinson's Disease
Source: PLoS One. 2014 Jan 8;9(1):e85305. doi: 10.1371/journal.pone.0085305 (PMC3885701; doi:10.1371/journal.pone.0085305)
Supplement: Table S1 — List of primers used for qPCR assays. (DOC) [file pone.0085305.s003.doc]

**Table S1. List of primers used for qPCR assays.**

| **Apoptosis mediators** | | |
| --- | --- | --- |
|  | **Forward (5' → 3')** | **Reverse (5' → 3')** |
| *egl-1* | CTAGCAGCAATGTGCGATGAC | GGAAGCATGGGCCGAGTAG |
| *ced-9* | TGCTCAGGACTTGCCATCAC | TTGACTCTCCGATGGACATTCTT |
| *ced-4* | AAGTCGAGGATTAGTCGGTGTTG | AGAGCCATTGCGAGTGACTTG |
| *ced-3* | TCAACGCGGCAAATGCT | GCCTGCACAAAAACGATTTTC |
| **Proteasome subunits** | | |
|  | **Forward (5' → 3')** | **Reverse (5' → 3')** |
| *pas-1* | GGCTGATCTCAACCAGTATTACACA | GAACAAAAGAGCACATCCCAAAC |
| *pas-2* | CGGCCGTAATGCAGGAATAT | AAGAAGCGATGCTCCAAACG |
| *pas-3* | CGGAGAGGAAATGCCAGTTG | ACGGTCTCTTTCCTCCAATCTG |
| *pas-4* | GTCGTACCACCAGGATCACAAA | TGCTGCAGCTCATCATTAACTTTT |
| *pas-5* | CAACATATTGGCGTCACATTCG | TGCCCGTTCGACCAGAGT |
| *pas-6* | CGAGAAATCAACTCCGGAACA | AAGTGTGTCGCGAAGAGCAA |
| *pas-7* | TTTCCAAGTCGAGTACGCTCAA | TTGCCACGAATTGCAATCAT |
| *pbs-1* | TCAGCACTGGAACCACTCTCA | TCGGTTCCGACGACAACTC |
| *pbs-2* | ATTTTGGAGCGTGATTTTAAGGTT | GGCGCGTTGGACAAGCT |
| *pbs-3* | GCTCCACGCGATTTCGTT | GCGCCAGAAGTTTTCACAAAC |
| *pbs-4* | GGGCAACAGCCGTACTTGTT | CGATCCATAATGGCATAGCAGAA |
| *pbs-5* | CTGCAATTTGTGCCACATCAC | TCACGTCCATTGGTGGAAGA |
| *pbs-6* | GATATGAGCGTCCGGAACTCA | ACGGAACGAATCCTTCATCAA |
| *pbs-7* | CTCTACGCCAAACGTTGCAA | ACTCCGGCGACAACAAGTG |
| *rpt-1* | TGGAAACATCAAGGTGCTTATGG | CTCATGAGAGCGGGATCGA |
| *rpt-2* | CCTGACGCCGCTAGCAAA | GCAACTTCAGACGGCATCTG |
| *rpt-3* | TGGAGAAGGACCACGAATGG | GATGGGCTGTTTTCCTTTGC |
| *rpt-4* | GTCAAGTTGTCCGACGGATTC | TGGCAAACATTCCAGCTTCTG |
| *rpt-5* | GAAGATGAATGTCAACAAGGATGTAAA | TGCATTGTGCTCCGTTGAAG |
| *rpt-6* | CCGAAGAATCCGATGAGAAAAC | CACTTTTTGCTGCGCATCA |
| *rpn-1* | CGGAAAGCCAAAGACAATCAC | GAGATATTCATCGTTCGCCAACT |
| *rpn-2* | TGACATTGTTGAACAGATGGAGATC | TGCGGCTGCGTTTGAA |
| *rpn-3* | ATACATTGTGGCGAAGGCTATTG | TGTACCGAGGTCCATCACGAA |
| *rpn-5* | GGAGAGCACAACATGCGTATGA | CAGCGAGACGTTCGAAAGTG |
| *rpn-6* | AATATTGGAAAAGCACCTGAAATGT | TTTGATGTGGAAGTGAAGTCATTGT |
| *rpn-7* | TCATTCAGTTGGCCGCTCTT | TGTGGCGATAGATAGCGATCAA |
| *rpn-8* | TCAGGAAGTTCACGATGATGGA | TCTGAAGGCACATGCTCGAA |
| *rpn-9* | GGGTGCAGCCAAGAGTTTTAGA | GGAGTTGACATCGTTCCTCCAT |
| *rpn-10* | AGTACTATGATTTGTGTCGACAATTCG | GGAGCCGAGTTGGTTGGAA |
| *rpn-11* | ACGTTTTCGCTATGCCACAGT | TGGATCGACCGCTTCGA |
| *rpn-12* | CAAAGGAGCCAAAAGATCTTGTC | CACTGAGAACCTTCGTCAACTCA |
| **House keeping genes** | | |
|  | **Forward (5' → 3')** | **Reverse (5' → 3')** |
| *cdc-42* | CTGCTGGACAGGAAGATTACG | CTCGGACATTCTCGAATGAAG |
| *pmp-3* | GTTCCCGTGTTCATCACTCAT | ACACCGTCGAGAAGCTGTAGA |
| *Y45F10D.4* | GTCGCTTCAAATCAGTTCAGC | GTTCTTGTCAAGTGATCCGACA |
